# Engineering secondary cell wall deposition in plants

**DOI:** 10.1111/pbi.12016

**Published:** 2012-11-12

**Authors:** Fan Yang, Prajakta Mitra, Ling Zhang, Lina Prak, Yves Verhertbruggen, Jin-Sun Kim, Lan Sun, Kejian Zheng, Kexuan Tang, Manfred Auer, Henrik V Scheller, Dominique Loqué

**Affiliations:** 1 Joint BioEnergy Institute, Physical Biosciences Division, Lawrence Berkeley National LaboratoryOne Cyclotron Road, Berkeley, CA, USA; 2 FSN Plant Biotechnology R&D Centre, Shanghai Jiaotong UniversityShanghai, China

**Keywords:** artificial positive feedback loop, biofuels, cell wall, lignin, saccharification, synthetic biology

## Abstract

Lignocellulosic biomass was used for thousands of years as animal feed and is now considered a great sugar source for biofuels production. It is composed mostly of secondary cell walls built with polysaccharide polymers that are embedded in lignin to reinforce the cell wall structure and maintain its integrity. Lignin is the primary material responsible for biomass recalcitrance to enzymatic hydrolysis. During plant development, deep reductions of lignin cause growth defects and often correlate with the loss of vessel integrity that adversely affects water and nutrient transport in plants. The work presented here describes a new approach to decrease lignin content while preventing vessel collapse and introduces a new strategy to boost transcription factor expression in native tissues. We used synthetic biology tools in *Arabidopsis* to rewire the secondary cell network by changing promoter-coding sequence associations. The result was a reduction in lignin and an increase in polysaccharide depositions in fibre cells. The promoter of a key lignin gene, *C4H,* was replaced by the vessel-specific promoter of transcription factor *VND6*. This rewired lignin biosynthesis specifically for vessel formation while disconnecting *C4H* expression from the fibre regulatory network. Secondly, the promoter of the *IRX8* gene, secondary cell wall glycosyltransferase, was used to express a new copy of the fibre transcription factor *NST1*, and as the *IRX8* promoter is induced by *NST1*, this also created an artificial positive feedback loop (APFL). The combination of strategies—lignin rewiring with APFL insertion—enhances polysaccharide deposition in stems without over-lignifying them, resulting in higher sugar yields after enzymatic hydrolysis.

## Introduction

Plant cell walls are virtually the only source of cellulose for the paper industry and will be a great source of sugars for the predicted lignocellulosic biofuels era (Carroll and Somerville, 2009; Simmons *et al*., 2008; Somerville *et al*., 2010). The utilization of plants to convert solar energy into transportable and storable energy will have positive impacts on the environment. It can help to reduce drastically the utilization of fossil-derived fuels, which will reduce carbon emissions into the atmosphere. Despite the environmental benefits of lignocellulosic biofuels, their production cost is unaffordably high. The raw sugar derived from plant cell walls is too expensive when compared to the price of crude oil. The main contributors to the high cost of cell wall–derived glucose are low sugar density of the biomass, cell wall recalcitrance to enzymatic hydrolysis and medium content in cellulose. Each factor either impacts transportation or requires intensive use of energy and chemicals for processing (Blanch *et al*., 2008; Klein-Marcuschamer *et al*., 2010; Searcy *et al*., 2007). Therefore, enhancement of polysaccharide accumulation in raw biomass and improvement of biomass digestibility will have important beneficial impacts on the cost of lignocellulosic biofuels production (Blanch *et al*., 2011; Klein-Marcuschamer *et al*., 2010).

By embedding the polysaccharide polymers and reducing their extractability and accessibility to hydrolytic enzymes, lignin is the major contributor to cell wall recalcitrance. There is usually a high negative correlation between lignin content and saccharification efficiency of plant cell walls (Chen and Dixon, 2007; Jorgensen *et al*., 2007; Vinzant *et al*., 1997). Unfortunately, most efforts to reduce lignin content during plant development resulted in severe biomass yield reduction particularly in dicotyledonous species (Franke *et al*., 2002; Shadle *et al*., 2007; Voelker *et al*., 2010), and therefore, there are very few crops exhibiting high lignin reduction. This relationship between secondary cell wall modification and plant growth is not unique to lignin modification, but is often correlated with loss of cell wall integrity causing vessel collapse as it is also observed when secondary cell wall genes involved in hemicellulose or cellulose biosynthesis are defective (Anterola and Lewis, 2002; Brown *et al*., 2005; Voelker *et al*., 2010). Vessels are essential for providing aboveground tissues with water and nutrients absorbed by the root system (Boyce *et al*., 2004; Déjardin *et al*., 2010; Gomez *et al*., 2008). However, lignin-related growth inhibition is not always related to vasculature collapse (Li and Chapple, 2010), it can be caused by constitutive induction of defence mechanism. Repression of the HCT enzyme (hydroxycinnamoyl CoA/shikimate hydroxycinnamoyl transferase) from the lignin biosynthesis pathway in *Arabidopsis* and alfalfa was showed to constitutively induce defence response and inhibit plant development, and both phenotypes were overcome by blocking the accumulation of the defence hormone salicylic acid (Gallego-Giraldo *et al*., 2011a,b). Hence, when silencing strategies are used to reduce lignin content in plants, the levels of gene repression to avoid biomass yield reduction are compromised.

Because woody biomass is mostly composed of secondary cell walls, strategies that increase cell wall thickness will increase biomass density. Such strategies would reduce transportation costs, which are significant contributors to the price of biomass delivered to the biorefinery (Aden *et al*., 2002; Kumar *et al*., 2005; Searcy *et al*., 2007). Furthermore, these improvements could also be used to reinforce stem strength to reduce lodging and increase wood quality for construction. However, to avoid undesired growth phenotypes, an increase in cell wall deposition needs to be developed cautiously and has to be designed to target specific cell types such as fibre and pith cells. It is well known, for example, that overexpression of secondary cell wall transcription factors is often associated with ectopic cell wall thickening and lignification. This has a negative effect on expanding cells and photosynthetic tissues, which is deleterious for plant growth (Goicoechea *et al*., 2005; Mitsuda *et al*., 2005; Zhong *et al*., 2008, 2011a,b). Recently, a transcription factor, belonging to the WRKY family, was isolated and shown to act as repressor of secondary cell wall deposition in the pith, and more interestingly, its repression induces thickening of the wall in the pith without impacting plant development (Wang and Dixon, 2011; Wang *et al*., 2011).

**Figure 1:**
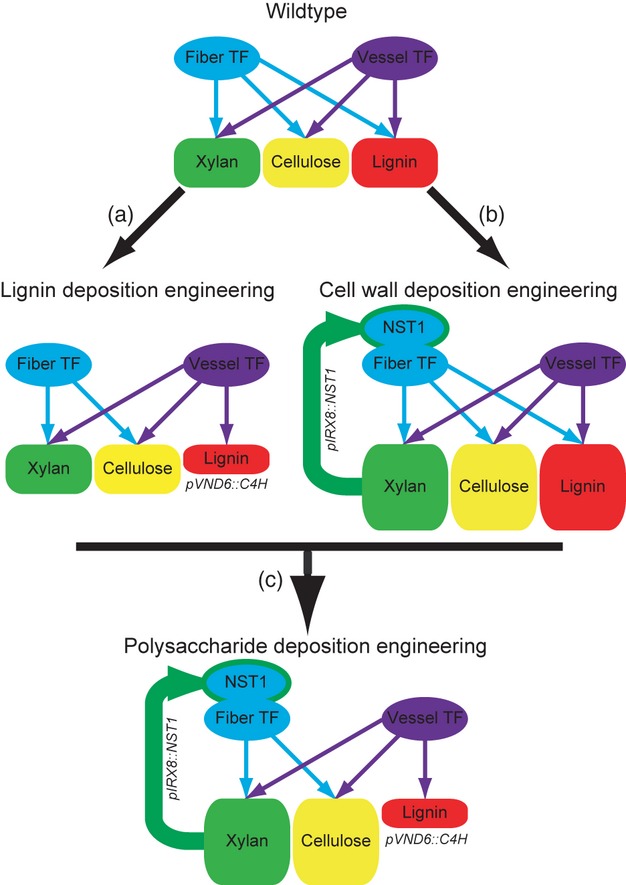
Model of secondary cell wall engineering (a) Lignin engineering: Lignin biosynthesis is dependent of the *C4H* gene expression (Figure S1), which is under the control of a vessel-specific promoter (*pVND6*), a promoter that is independent of the fibre regulatory network. (b) Cell wall artificial positive feedback loop (APFL): The *IRX8* secondary cell wall promoter (*pIRX8*), known to be one of the downstream induced targets of the *NST1* transcription network, was used to express a new copy of *NST1*. When secondary cell wall transcription factors (comprising *NST1*) are expressed, they induce their downstream network and *pIRX8* used to control the expression of the new *NST1* allele. Consequently, it will further enhance the accumulation of NST1 transcription factor, which over-induce secondary cell wall gene expression including *pIRX8* promoter, causing an increase in secondary cell wall deposition compared to wild-type plants. (c) The APFL was combined with the lignin engineering, allowing enhancing preferably polysaccharide deposition, because lignin biosynthesis is under control of *pVND6,* which is not regulated by *NST1*.

Secondary cell wall regulatory network is now partially understood and seems to be conserved across many species from dicot to monocot plants (Christiansen *et al*., 2011; Handakumbura and Hazen, 2012; Ruprecht *et al*., 2011). Interestingly, cell differentiation into vessel or fibre cells starts independently. Both are regulated by independent master transcription factors that rapidly share the same regulatory network to control the expression of many genes involved in the biosynthesis of the three major secondary cell wall components: cellulose, xylan and lignin (Cano-Delgado *et al*., 2010; Ohtani *et al*., 2011; Zhong *et al*., 2010, 2011b). This makes it challenging to manipulate cell wall composition or content of woody tissues without impacting cell wall integrity and plant development (Anterola and Lewis, 2002; Brown *et al*., 2005; Voelker *et al*., 2010). In this study, synthetic biology tools were used to rewire part of the secondary cell wall network and to develop two new and complementary strategies to manipulate cell wall biosynthesis in specific tissues. This strategy was designed to reduce lignin content in fibre cells (and thus cell wall recalcitrance) and to enhance polysaccharide deposition in fibres without impacting plant development. We first rewired the regulation of lignin biosynthesis by disconnecting it from many regulatory networks including that of the fibre and largely restricted its control to the one of vessels (Figure 1a). Next, to enhance secondary cell wall deposition in specific cell types, we created an artificial positive feedback loop (APFL) to boost the expression of the NST1 master transcription factor controlling secondary cell wall biosynthesis in fibres (Figure 1b). We applied this APFL to the low-lignin plants, engineered with a lignin biosynthesis that is disconnected from the fibre secondary cell wall regulatory network (Figure 1c). This engineering allowed us to generate healthy plants with reduced lignin and enhanced cell wall deposition, which—after various pretreatments—exhibit improved sugar releases from enzymatic hydrolysis as compared to wild type.

## Results

### Characterization of vessel-specific promoter *pVND6*

Because of the importance of vessels for transport of water and nutrients to photosynthetic organs, integrity of this tissue is required for good plant development. Both transcription factors, *VND6* and *VND7,* have been characterized as master regulators for vessel formation, suggesting that they have a vessel-restricted expression pattern and would be independent of those that regulate fibre development (Kubo *et al*., 2005; Yamaguchi *et al*., 2008, 2010). To correlate the spatio-temporal activity of the promoter controlling the expression of these transcription factors with lignin biosynthesis, the promoter *VND6* (*pVND6*) was used to express *CADd* to complement the *cad-c cad-d* mutant (Sibout *et al*., 2005, Figure S1a). The *cad-c cad-d* mutant was selected as a pre-screen tool because a homozygous mutant can be easily grown and displays an easily observable phenotype corresponding to partial vessel collapse as well as cell wall redness caused by the accumulation of a side-product derived from the accumulation of hydroxycinnamaldehydes. Stem cross-sections of two independent *cad-c cad-d* + *pVND6::CADd* lines were analyzed under bright light (Figure S1a). The reduction in redness seen particularly in xylem and the restoration of the vessel integrity observed for both *cad-c cad-d* + *pVND6::CADd* lines met the acceptance criteria for use of this promoter in further investigations.

To compare the strength of *VND6* and *C4H* promoters (*pVND6* and *pC4H*, respectively), both were used to express the *F5H1* gene in *f5h1-1* null mutant background (Meyer *et al*., 1998). The use of this mutant as a tool to study the activity of these promoters was based on the absence of growth phenotype, vessel collapse, and sinapyl alcohol unit in the lignin that are easily detected by Mäule staining (Nakano *et al*., 1992). Two independent lines for both promoters were selected for analysis, and the activity of each promoter was compared by the amount of sinapyl alcohol units incorporated into the lignin using Mäule staining as the readout (Figure S1b). Cross-sections of stems from both lines expressing the *F5H1* gene under the control of *pVND6* show a much lower level of red coloration after the Mäule staining than that of the lines expressing *F5H1* with *pC4H*. The lower coloration is more pronounced in interfascicular fibres of *f5h1-1* + *pVND6::F5H1* lines compared to those of *f5h1-1* + *pC4H::F5H1* lines and is caused by a lower accumulation of sinapyl alcohol. This observation supports that *pVND6* activity is largely restricted to vessel cells in contrast to that of *pC4H*. Taken together with the *cad* complementation (Figure S1), these data demonstrate that *pVND6* is a suitable promoter to manipulate lignin biosynthesis in vessels.

### Restriction of lignin biosynthesis to vessels

**Figure 2:**
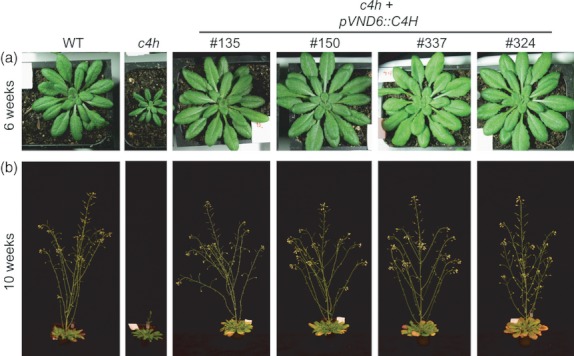
Pictures of lignin-engineered plant 6- (a) and 10-week-old (b) wild-type (WT), *c4h* mutant, and four *c4h* + *pVND6::C4H* complemented lines (135, 150, 324, 337). Plants were grown together on soil under short-day conditions for 5 weeks (10 h/14 h light/dark cycle) prior to be transferred under long-day growth conditions (14 h/10 h light/dark cycle) until maturity.

Lignin biosynthesis pathway is well characterized and mutations in this pathway, particularly in the genes involved in the earlier enzymatic steps, affect drastically plant growth and fertility. Therefore, controlling the expression of one of these genes should be sufficient to control the entire production of monolignols. We selected the *C4H* gene, encoding for the second enzyme in the lignin biosynthesis pathway (Figure S2), as a target gene to control the flux through that pathway and consequently the production of monolignols. To control the expression of *C4H*, we used the *c4h* mutant (Ruegger and Chapple, 2001; Schilmiller *et al*., 2009) and transformed the heterozygote line (due to the sterility of the homozygous line) with the *pVND6::C4H* gene construct. Several transformants harbouring the *pVND6::C4H* fragment were selected and genotyped for the presence of homozygous *c4h* allele and four independent lines were used for further analysis. In contrast to the non-transformed *c4h* homozygous plants, the *c4h* + *pVND6::C4H* plants did not show any obvious growth difference when compared to wild-type plants (WT). The *c4h* + *pVND6::C4H* plants were fertile and able to generate large rosettes and tall stems (Figure 2). However, old leaves from *c4h* + *pVND6::C4H* plants showed anthocyanin accumulation only in leaf vasculatures in contrast to those of wild-type plants that turned completely purple (data not shown), evidence that the activity of the *pVND6* is more restricted than that of the native *C4H* promoter.

**Figure 3:**
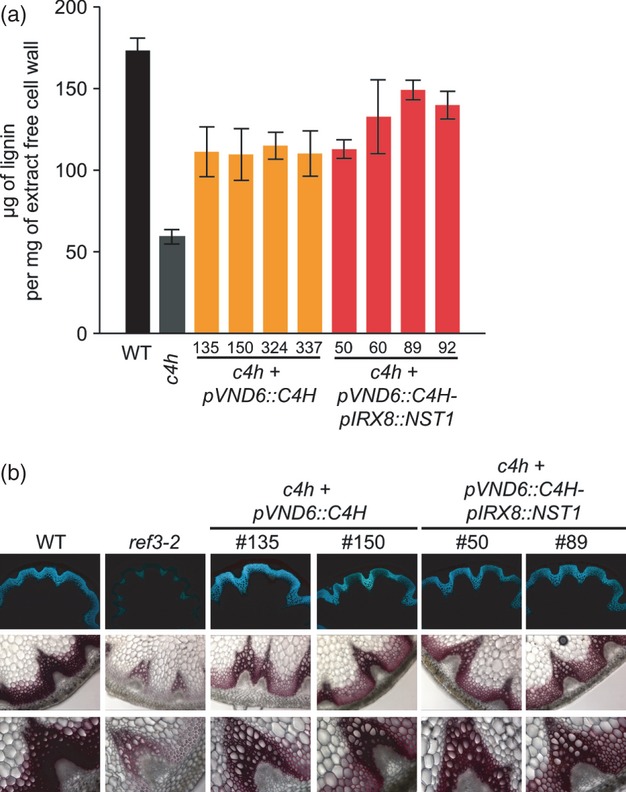
Impact of the cell wall engineering on lignin deposition in stems (a) Lignin content was determined from extract-free mature stems derived from wild type (WT), *c4h* mutant, four *c4h* + *pVND6::C4H* lines (135, 150, 324, 337) and four *c4h* + *pVND6::C4H* lines harbouring the artificial positive feedback loop (APFL) *pIRX8::NST1* construct (50, 60, 89, 92). (b) Stem cross-sections from wild-type (WT), *c4h* mutant, two *c4h* + *pVND6::C4H* (135, 150) and two *c4h* + *pVND6::C4H* lines harbouring the APFL *pIRX8::NST1* construct (60, 89) under UV (top panel) and under bright light after phloroglucinol–HCl staining (middle and bottom panels).

Using the acetyl bromide method, analysis of lignin content in senesced stems from several *c4h* + *pVND6::C4H* lines shows that it was approximately 2/3 that of the wild type (Figure 3a). To verify the lignin distribution in the stems, stem cross-sections were stained with phloroglucinol–HCl reagents. Cross-sections of both *c4h* + *pVND6::C4H* lines showed reduction of lignin in the interfascicular fibres compared to wild-type plants. In contrast to that of the homozygous *c4h* mutant, xylem tissues of *c4h* + *pVND6::C4H* lines show a strong purple coloration after phloroglucinol staining and no vessel collapse, which is similar to what is observed in wild-type plants (Figure 3b).

### Design of an APFL to overexpress *NST1*

The transcriptional network controlling secondary cell wall deposition in vessel and fibres has already been well investigated. Several transcription factors were identified as master switches for secondary cell wall deposition in vessel or fibre cells, but all regulate virtually the same downstream network by controlling the expression of the main secondary genes involved in the biosynthesis of cellulose, hemicelluloses, and lignin polymers. Thus, these master switches are potential targets for manipulation of cell wall thickness (Mitsuda and Ohme-Takagi, 2009). Unfortunately, without tight expression control they are more harmful to the plant. Several groups have shown that expressing them with a constitutive 35S promoter causes ectopic secondary cell wall, thus inhibiting plant growth (Goicoechea *et al*., 2005; Mitsuda *et al*., 2005; Yamaguchi *et al*., 2010; Zhong *et al*., 2008).

To develop a tighter over-expression system, we designed an APFL (Figure 1b) by expressing a new copy of a master transcription factor under the control of one of its downstream induced promoters. The hypothesis was that this would enhance the overall expression of the master transcription factor because, when its native promoter turns on, it would also induce the expression of its downstream targets—including the APFL that will produce more of the master transcription factor. To verify this hypothesis, we tested for enhanced cell wall thickening. We selected the *NST1* transcription factor that controls secondary cell wall deposition in fibres and used the promoter (*pIRX8*) of the secondary cell wall *IRX8* glycosyltransferase gene that is known to be induced by *NST1* (Mitsuda *et al*., 2005; Zhong *et al*., 2010). As it was also known that the *NST1* transcription factor positively controls the lignin biosynthetic pathway but does not control the activity of *pVND6* (used to control lignin biosynthesis in the *c4h* + *pVND6::C4H*), we transformed some *c4h* + *pVND6::C4H* plants (line 135) with the *pIRX8::NST1* gene construct to generate new transgenic *Arabidopsis* lines *c4h* + *pVND6::C4H-pIRX8::NST1*. The lignin-engineered lines (*c4h* + *pVND6::C4H*) were selected as the genetic background to disconnect the overexpression of polysaccharide biosynthesis from lignin biosynthesis (Figure 1c).

**Figure 4:**
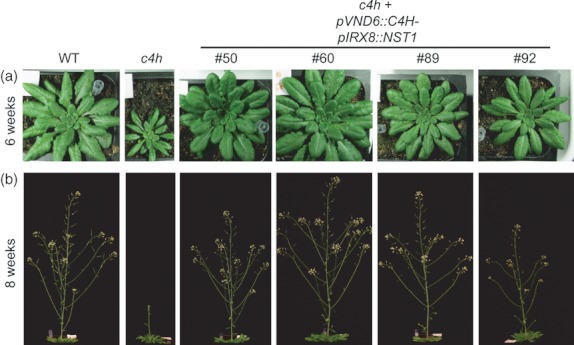
Pictures of cell wall engineered plants Six- (a) and 8-week-old (b) wild-type (WT), *c4h* mutant, and four *c4h* + *pVND6::C4H* lines harbouring the artificial positive feedback loop *pIRX8::NST1* construct (50, 60, 89, 92). Plants were grown together on soil under short-day conditions for 5 weeks (10 h/14 h light/dark cycle) prior to be transferred under long-day growth conditions (14 h/10 h light/dark cycle) until maturity.

Several *c4h* + *pVND6::C4H-pIRX8::NST1* lines were generated and did not show any obvious growth difference when compared to wild type and *c4h* + *pVND6::C4H* plants (Figure 4). The *c4h* + *pVND6::C4H-pIRX8::NST1* lines were fertile and able to generate large rosettes and tall stems. Like those of *c4h* + *pVND6::C4H* old leaves, leaf vasculatures from the *c4h* + *pVND6::C4H-pIRX8::NST1* lines were purpled as a result of anthocyanin accumulation in contrast to wild-type old leaves that turned completely purple (data not shown). Expression analysis of both *NST1* alleles (native and APFL allele) was verified by semi-quantitative RT-PCR and suggests that the native *NST1* is expressed at the same level in wild-type plants and both *c4h* + *pVND6::C4H* and *c4h* + *pVND6::C4H-pIRX8::NST1* lines (Figure S3). Expression of the new *NST1* allele was only detected in the *c4h* + *pVND6::C4H-pIRX8::NST1* lines, resulting in a higher general expression level of the *NST1* gene (native and APFL allele) in *c4h* + *pVND6::C4H-pIRX8::NST1* stems (Figure S3).

### Artificial positive feedback loop increases cell wall deposition in fibre cells

To verify the impact of *NST1* overexpression on lignin deposition, we first quantified lignin content in mature stems from the different engineered lines using the acetyl bromide method (Figure 3a). The *c4h* + *pVND6::C4H-pIRX8::NST1* lines exhibited a slight increase in lignin content compared to the *c4h* + *pVND6::C4H* plants (parent line 135), but that level remained lower than that of wild type. This analysis was extended with stem cross-sections that were stained with phloroglucinol–HCl. Cross-sections of the *c4h* + *pVND6::C4H-pIRX8::NST1* lines (60 and 89) show no vessel collapse and less lignin in the interfascicular fibres compared to wild type (Figure 3b).

**Figure 5:**
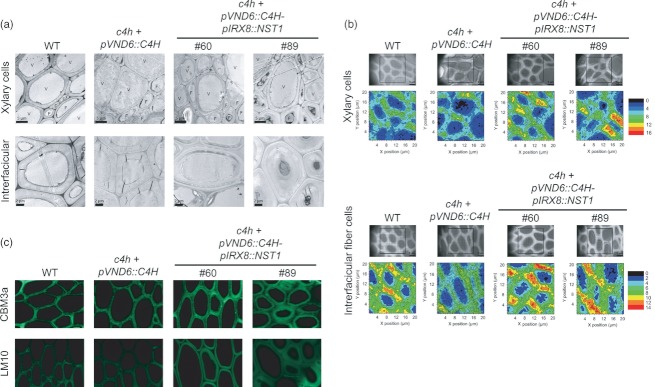
Impact of the cell wall engineering on polysaccharide deposition in stems Stem cross-sections analysis from wild type (WT), *c4h* + *pVND6::C4H* line (135), and two *c4h* + *pVND6::C4H* lines harbouring the artificial positive feedback loop *pIRX8::NST1* construct (60, 89). (a) TEM images, top panel represents xylary area with vessel and fiber cells (V and F, respectively), and bottom panel represents interfascicular area with fibre cells only. (b) Cellulose map defined by confocal Raman microspectroscopy of fibre cells from xylary area (first top panel: bright-field image, second top panel: cellulose map) and of interfascicular fibres (third top panel: bright-field image; fourth top panel: cellulose map). (c) Immunofluorescence micrographs of interfascicular fibre cells labelled with CBM3a for crystalline cellulose (top panel) and LM10 for xylan (bottom panel).

The next step was to analyze the impact of *NST1* overexpression on cell wall deposition. Cell wall thickening was analyzed by transmission electron microscopy on stem cross-sections (Figure 5a). Compared to those of wild type and the *c4h* + *pVND6::C4H* line, fibre cells (xylary and interfascicular) from the *c4h* + *pVND6::C4H-pIRX8::NST1* lines (line 60 and 89) showed an increase in cell wall deposition, although the cell wall thickening is more moderate and irregular for the *c4h* + *pVND6::C4H-pIRX8::NST1* line 60 than for line 89 (Table S1). In contrast, no significant difference was observed for the vessel cells between the wild type and the different engineered lines. Furthermore, we measured stem diameters and weights of the engineered lines and found that both *c4h* + *pVND6::C4H-pIRX8::*NST1 lines exhibit higher biomass density (>10%) than the parental *c4h* + *pVND6::C4H* line (Table S2). To determine whether the increase was correlated to increased polysaccharide deposition, we further investigated *c4h* + *pVND6::C4H-pIRX8::NST1* lines using confocal Raman microspectroscopy and immunofluorescence (Figures 5b,c). Cellulose distribution was analyzed by confocal Raman microspectroscopy by integrating the area between 1070 and 1140/cm of the collected Raman spectra at defined positions, allowing us to draw a cellulose map for different fibre cells (Figure 5b). As a result of *NST1* overexpression, the *c4h* + *pVND6::C4H-pIRX8::NST1* line showed thicker and denser cellulose distribution (represented by a higher intensity) compared to that of the wild type and *c4h* + *pVND6::C4H* lines in both xylary and interfascicular fibres. Surprisingly, the *c4h* + *pVND6::C4H* line displayed a lower cellulose density than wild type, particularly in its interfascicular fibres.

Immunofluorescence analysis was used as an alternative approach to analyze cellulose and xylan deposition in interfascicular fibre cells using CBM3a and mAb-LM10, respectively, on stem cross-sections of wild type, *c4h* + *pVND6::C4H*, and two (lines 60 and 89) of the *c4h* + *pVND6::C4H-pIRX8::NST1* lines (Figure 5c). CBM3a labelling analysis reveals that wall thickness was enhanced with crystalline cellulose for both *c4h* + *pVND6::C4H-pIRX8::NST1* lines compared to that of the wild type and *c4h* + *pVND6::C4H* line. The increased cellulose deposition observed in *c4h* + *pVND6::C4H-pIRX8::NST1* plants was more pronounced in line 89 than in line 60. The mAb-LM10 labelling analysis reveals that wall thickening is also correlated with an increase in xylan deposition, especially for the *c4h* + *pVND6::C4H-pIRX8::NST1* line 89 when compared with that of the wild type and *c4h* + *pVND6::C4H* line. In contrast, the *c4h* + *pVND6::C4H-pIRX8::NST1* line 60 shows a more moderate and irregular increase.

### Impact of secondary cell wall engineering on saccharification efficiency

**Figure 6:**
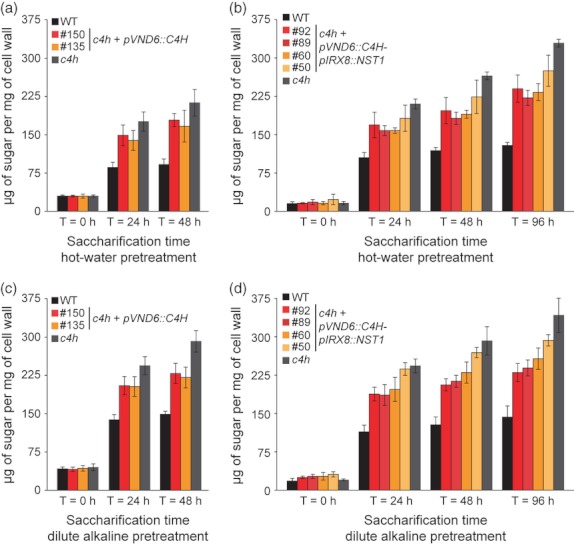
Saccharification efficiency of biomass derived from engineered plants Amount of sugars released from enzymatic digestion (after 24, 48 or 96 h) of mature stems derived wild-type (WT), *c4h* mutant, two *c4h* + *pVND6::C4H* lines (135, 150), and four *c4h* + *pVND6::C4H* lines harbouring the artificial positive feedback loop *pIRX8::NST1* construct (50, 60, 89, 92) after hot water (a, b) or dilute alkaline (c, d) pretreatments.

Cell wall analysis of both engineered lines *c4h* + *pVND6::C4H* and *c4h* + *pVND6::C4H-pIRX8::NST1* revealed that lignin content was reduced and the cell wall content in the *c4h* + *pVND6::C4H-pIRX8::NST1* line was increased in comparison with wild type, suggesting that biomass for the engineered lines should be less recalcitrant to enzymatic hydrolysis. Therefore, ball-milled stems from wild type, *c4h*, *c4h* + *pVND6::C4H* and *c4h* + *pVND6::C4H-pIRX8::NST1* plants were subjected to saccharification after two different mild pretreatments (hot water and dilute alkali), and the amount of sugar liberated was measured after 24, 48 and 96 h (Figures 6 and S4). Results showed that in both pretreatments, sugar release was faster and much higher for the cell wall engineered plants than for wild type and was almost as good as a the *c4h* lignin mutant. For each time point, the sugar released from stems of *c4h* + *pVND6::C4H-pIRX8::NST1* lines was higher than that from the parental line (*c4h* + *pVND6::C4H* line 135). It was more than 2.5 times higher than that from wild type (Figure S4) at 96 h after hot water pretreatment and two times higher than that from wild-type stems (Figure S4) at 96 h after dilute alkaline pretreatment.

## Discussion

Modifying the lignin content has always been a challenge in crops or trees, because the more severe the reduction, the more biomass yield is negatively affected. This reduction is also often associated with a loss of integrity in vessels, the tissues that are responsible for water and nutrient distribution from roots to the aboveground organs (Boyce *et al*., 2004; Déjardin *et al*., 2010; Gomez *et al*., 2008). Lignin is considered to be the main inhibitory factor for pulping, forage digestibility and efficient enzymatic hydrolysis of plant cell wall polysaccharides, but it cannot be easily removed (Chen and Dixon, 2007; Jorgensen *et al*., 2007; Vinzant *et al*., 1997). Therefore, the present strategy focused on reducing lignin in tissues other than vessels, so that vessel integrity is maintained. In addition, the strategy sought to disconnect lignin biosynthesis from NST1, one of the key transcription factor switches controlling secondary cell wall deposition in fibre cells, allows manipulation of its expression without affecting lignin biosynthesis. By re-engineering few control points of the secondary cell wall biosynthesis (Figure 1), we demonstrated that we are able to reduce the lignin content and to increase cell wall thickening in fibres without obvious alteration of plant development (Figures 5). We replaced the promoter driving the expression of the *C4H* gene, which controls a key step in lignin biosynthesis, by that of the *VDN6* transcription factor (*pVND6*). The activity of *pVND6* is largely restricted to vessel cells (Kubo *et al*., 2005; Zhong *et al*., 2008), allowing a preferable spatio-temporal control of lignin deposition (Figures 3 and S1). This suggests that the *pVND6* promoter could be replaced by several other vessel-specific promoters, such as *pVND7*, *pVNI*, *pMC9* and *pACL5* (Bollhöner *et al*., 2012; Ko *et al*., 2012; Kubo *et al*., 2005; Vera-Sirera *et al*., 2010; Yamaguchi *et al*., 2010). In addition, the control of the lignin biosynthesis could also be performed by controlling the expression of PAL, 4CL1 or C3'H enzyme instead of C4H; as several lignin mutants from various plant species, affected for one these enzymes, exhibit lignin content reduction, vessel collapse and growth defect phenotypes (Anterola and Lewis, 2002; Brown *et al*., 2005; Voelker *et al*., 2010).

To transfer the lignin rewiring approach to crops, lignin mutants in a target crop need to be isolated or generated via a gene-silencing approach (Schwab *et al*., 2006; Voinnet *et al*., 1998). In these mutants, lignin biosynthesis will be restored in the vessels by using a vessel-specific promoter from the target crop or related species to express a new allele encoding a protein that exhibits the same function as the repressed protein. This new allele should have a different sequence than the silenced gene to be protected against silencing. This can be achieved by using an allele from a different plant species or via gene synthesis. For example, a new *4CL* or C3′H encoding sequence could be expressed with a vessel-specific poplar promoter (Bollhöner *et al*., 2012; Ko *et al*., 2012) in *4CL* antisense or C3′H RNAi poplar lines respectively to restore cell wall integrity and function of the vessels (Coleman *et al*., 2008a,b; Kitin *et al*., 2010; Voelker *et al*., 2010). Such engineering should restore growth and increase biomass yield of the silenced lines and retain great saccharification efficiency. Alternatively, the defective enzymatic step could be bypassed with alternative routes to synthesize the missing precursors. For example, the *SmF5H* gene from *Selaginella* could be expressed with a vessel-specific promoter to restore the integrity of vessel and plant growth of the C3′H RNAi poplar lines (Coleman *et al*., 2008a,b). This *SmF5H* gene was recently shown in *Arabidopsis* to be able to restore the growth of *hct-* and c3′′h-deficient mutants lacking the ability to produce p-coumaroyl shikimate and to meta-hydroxylate p-coumaroyl shikimate, respectively (Figure S2; Weng *et al*., 2010).

Another challenge was to manipulate general secondary cell wall biosynthesis using transcription factors to increase polysaccharide deposition without causing deleterious side-effects and over-lignification of the plant (Goicoechea *et al*., 2005; Mitsuda *et al*., 2005; Yamaguchi *et al*., 2010; Zhong *et al*., 2008, 2011a). The creation of an APFL using a secondary cell-wall-specific promoter (*pIRX8* in this study) and the *NST1* transcription factor allowed us to increase secondary cell wall biosynthesis specifically in stems without altering plant development (Figures 1, 2 and 4). This APFL for the secondary cell wall is not restricted to the use of either *pIRX8* or *NST1*. Either could be replaced by different specific secondary cell wall promoters and transcription factors responsible for secondary cell wall deposition in fibre cells (Demura and Ye, 2010; Zhong *et al*., 2010). To our knowledge, this is the first APFL that has been developed in plants. There is only one example of an artificial negative feedback loop, which was developed in plants to delay senescence (Gan and Amasino, 1995). This negative feedback loop is based on the use of the early senescence-induced promoter (*pSAG12*) to express an *IPT* gene encoding for an isopentenyltransferase. It produces cytokinins at that specific developmental stage to delay senescence and keep the plant photosynthetically active much longer (Gan and Amasino, 1995). Furthermore, because of the conservation of senescence repression by cytokinins across plant species, this synthetic negative feedback loop was transferred into various crops (grasses and dicots) and was used to increase lifespan and improve plant biomass yield (Calderini *et al*., 2007; McCabe *et al*., 2001; Robson *et al*., 2004). Interestingly, the secondary cell wall regulatory network falls into the same category of conserved mechanisms across plant species (Christiansen *et al*., 2011; Handakumbura and Hazen, 2012; Ruprecht *et al*., 2011) and was validated by the demonstration that *NST1* like transcription factors from poplar and rice, were able to complement a double *nst1/nst3* mutant from *Arabidopsis* using the *NST1* promoter from *Arabidopsis* (Zhong and Ye, 2010; Zhong *et al*., 2011a). It demonstrates that poplar and rice *NST* proteins are able to regulate the same promoters as the *Arabidopsis NST* and control secondary cell wall network in *Arabidopsis*. Taken altogether, this strongly suggests that the APFL developed in *Arabidopsis* to overexpress *NST1* transcription factor in fibres could be rapidly implemented into other vascular plant species to enhance secondary cell wall deposition. Therefore, this APFL technology could be used to increase cell wall content in plants designated not only for bioenergy, the pulping industry and forage production, but also to reinforce stem strength to reduce crop lodging and associated seed losses.

In summary, we presented two compatible approaches: (i) to narrow down lignin biosynthesis into vessels and (ii) to increase secondary cell wall thickening. Both were used to generate healthy plants with increased sugar yield upon saccharification. These approaches should open new ways for crop optimization and should benefit to lignocellulosic biofuels, paper and forage industries. Furthermore, we believe that the approach used to develop this APFL should be applicable to other metabolic pathways controlled by master transcription factors to boost their own expression in native tissues.

## Experimental procedures

### Plant material and growth conditions

Wild-type *Arabidopsis thaliana* plant (ecotype Columbia), *c4h* mutants (ecotype Columbia; *ref3-2* mutant harbouring a poorly functional *C4H* allele; Ruegger and Chapple, 2001; Schilmiller *et al*., 2009), *c4h* + *pVND6::C4H* lines and *c4h* + *pVND6::C4H-pIRX8::NST1* lines were grown on soil from 8- to 10-day old seedling germinated either on soil or on sterile media. Because of its male sterility, the homozygote *c4h* mutants were identified by PCR-based genotyping from segregating populations derived from heterozygote *c4h* mutants. The *c4h* + *pVND6::C4H* lines were generated via floral dipping (Clough and Bent, 1998) of genotyped heterozygote *c4h* mutants with *Agrobacterium tumefaciens* GV3101 strain harbouring the *pA6-pVND6::C4H* binary vector. Selection of T1 and T2 *c4h* + *pVND6::C4H* lines was made on Murashige and Skoog medium supplemented with 1% sucrose, 1% agar and containing 30 μg/mL hygromycin followed by a *c4h* allele genotyping. Homozygote *c4h* mutants harbouring the *pVND6::C4H* DNA construct were named *c4h* + *pVND6::C4H* and used for downstream analysis or for agrobacterium-mediated transformation. The *c4h* + *pVND6::C4H-pIRX8::NST1* lines were generated from the parent *c4h* + *pVND6::C4H* line (line 135) via floral dipping with *Agrobacterium tumefaciens* GV3101 strain harbouring the *pKan-pIRX8::NST1* binary vector. Selection of T1 and T2 *c4h* + *pVND6::C4H-pIRX8::NST1* transgenic plants was made on Murashige and Skoog medium supplemented with 1% sucrose, 1% agar and containing 30 μg/mL hygromycin and 50 μg/mL kanamycin. The homozygote *c4h* allele was verified for each generation.

Plants designated for analysis were grown on soil under short-day condition for 5 weeks (10 h/14 h light/dark cycle) prior being transferred to long-day growth condition (14 h/10 h light/dark cycle) until maturity at 150 μmol/m^2^/s, 22 °C and 60% humidity. All the other plants were grown under long-day condition (14 h/10 h light/dark cycle) at 100 μmol/m^2^/s, 22 °C, and 55% humidity.

### Generation of *pA6-pVND6::C4H* and *pTkan-pIRX8::NST1* binary vectors

The *C4H* (REF3; At2g30490) and *NST1* (At2g46770) encoding DNA sequences were amplified from *Arabidopsis* cDNA using gene-specific primers extended with gateway b1 and b2 sequences as described in the Gateway manual (Life Technologies, Grand Island, NY). Both pairs *F-C4H-GWb1/R-C4H-GWb2* and *F-NST1-GWb1/R-NST1-GWb2* were used to clone *C4H* and *NST1* encoding DNA sequences respectively (Appendix S1) using Gateway technology (Life Technologies). The DNA fragments were introduced into the *pDONR221-f1* entry vector (Lalonde *et al*., 2010) by BP recombination to create *pDONR-F1-C4H* and *pDONR-F1-NST1*, sequence verified, then transferred in *pA6-pVND6-GW* and *pTkan-pIRX8-GW* (Appendix S1) by LR recombination to create *pA6-pVND6::C4H* and *pTkan-pIRX8::NST1* binary vectors, respectively.

### Genotyping of *c4h*, *c4h* + *pVND6::C4H* and *c4h* + *pVND6::C4H-pIRX8::NST1* plants

Genotyping was performed on purified genomic DNA (Appendix S1) extracted from wild type and plant harbouring the *c4h* allele and *pVND6::C4H* or *pIRX8::NST1* genes. The genotyping of the genomic *c4h* allele was performed by PCR using the *F-ref3-2/R-ref3-2* primer pair (Appendix S1) to amplify an 896 bp DNA fragment followed by a HinfI restriction digest because the *c4h* allele has lost the restriction site (Ruegger and Chapple, 2001; Schilmiller *et al*., 2009; the presence of a genomic wild-type allele was recognized by the generation of a double 625/271 bp fragment after restriction). The presence of *pVND6::C4H* and *pIRX8::NST1* transgenes was verified by PCR with the primer pairs *F1-pVND6/R1-C4H* and *F1-pIRX8/R1-NST1*, respectively (Appendix S1).

### Histochemical staining

For all analyzed lines, except the homzygote *c4h* mutant, base of equivalent primary stems (from approximately 20 cm tall plants and 8 cm for the homzygote *c4h* mutant) was embedded in 7% agarose before being transversally sectioned to a thickness of 100 μm using a vibratome (Leica VT1000S; Microsystems Inc., Buffalo Grove, IL). For bright-field and UV fluorescence analysis, sections were directly mounted in water. For Wiesner lignin staining (phloroglucinol–HCl staining), sections were incubated for 3 min in phloroglucinol–HCl 2% (w/v) solution composed of phloroglucinol (VWR International, Brisbane, CA) dissolved in a 2 : 1 mixture of ethanol and concentrated HCl and rinsed with water (Nakano *et al*., 1992). For Mäule lignin staining, sections were incubated in 0.5% KMnO_4_ for 2 min, rinsed with water several time until the dark purple solution is washed out, then incubated in 10% HCl for 1 min and mounted after the addition of a drop of aqueous ammonia (Nakano *et al*., 1992). All sections were analyzed using a bright-field/fluorescent microscope (Leica DM4000B; Microsystems Inc., Buffalo Grove, IL).

### Electron microscopy

Primary stems of wild-type, *c4h* + *pVND6::C4H* and *c4h* + *pVND6::C4H-pIRX8::NST1* plants were fixed in 4% glutaraldehyde and 1% paraformaldehyde in PBS buffer for 1 h under vacuum and placed on a rocker overnight for 12 h. After rinsing three times with PBS, samples were transferred in ice to be post-fixed in 1% OsO4, rinsed three times with PBS and dehydrated in an graded ethanol series followed by a final 100% acetone step employing a PELCO-Biowave microwave oven (Ted Pella, Inc., Redding, CA) equipped with a PELCO-ColdSpot heat sink (40 s on, 20 s off and 40 s on). Subsequently, samples were fixed by infiltration, embedded and polymerized in resin. Hardened blocks were sectioned at 100 nm thickness and images performed with an electron microscope (FEI Tecnai 12, FEI, Hillsboro, OR) operating at 120 kV and equipped with a 2k × 2k CCD camera (FEI).

### Confocal raman microspectroscopy

Analysis of cellulose deposition in primary stems of wild type, *c4h* + *pVND6::C4H* and *c4h* + *pVND6::C4H-pIRX8::NST1* plants was performed with a LabRam HR 800 confocal Raman system (Horiba Jobin Yvon, Edison, NJ) as described in Sun *et al*. (2011). Images were collected using a 785 nm diode laser and a high numerical aperture 100× (oil NA 1.40) objective to achieve a submicron spatial resolution. A 20 by 20 μm region was measured for each image with a mapping step of 0.5 μm, an integration time of 1 s and a spectral resolution of approximately 4/cm. A SWIFT mode was utilized for the raster mapping to significantly increase mapping speed. The LabSpec5 software (HORIBA Scientific, Edison, NJ) was used for setting up measurements and data processing. The spectra in the range of 1050–1150/cm were despiked and smoothed using the Savitsky–Golay algorithm. The spectra were then baseline corrected and further smoothed using Fourier smoothing coupled with cosine apodization function. The integrated intensity over the range of 1070–1140/cm of the processed spectra was used to generate the cellulose maps by OriginPro 8 (OriginLab, Northampton, MA).

### Immunolocalization

Base of equivalent primary stems of wild-type, *c4h* + *pVND6::C4H* and *c4h* + *pVND6::C4H-pIRX8::NST1* plants was embedded in LR white resin and transversely sectioned to a thickness of 1 μm with a Leica UC6 ultramicrome as described in Yin *et al*. (2011). Sections were labelled either with the anti-xylan LM10 monoclonal antibody (McCartney *et al*., 2005) or with CBM3a (a probe to crystalline cellulose; Blake *et al*., 2006) and analyzed using a fluorescent microscope (Leica DM4000B; Microsystems Inc.). Images were captured with Micropublisher Q-imaging camera coupled to Metamorph software (Molecular Devices, Sunnyvale, CA). With the LM10 antibody, the immunolabellings were carried out as described in Verhertbruggen *et al*. (2009). LM10 was provided as supernatant, used at a 10-fold dilution and the secondary antibody was an anti-rat coupled with FITC diluted 100-fold. The detection of crystalline cellulose with CBM3a was carried out as described in McCartney *et al*. (2004), and the primary and secondary antibodies were a mouse anti-HIS diluted 100-fold and an anti-mice coupled with FITC diluted 100-fold, respectively.

### Lignin quantification

Senesced stems of wild-type, homozygote *c4h* mutant, *c4h* + *pVND6::C4H* and *c4h* + *pVND6::C4H-pIRX8::NST1* plants were ball-milled using a Mixer Mill MM 400 (Retsch Inc., Newtown, PA) and stainless steel balls for 2 min at 30/s. Extract-free cell wall residues (CWR) were obtained by sequentially washing 50 mg of ball-milled stems with 1 mL of 96% ethanol at 95 °C twice for 30 min and vortexing with 1 mL of 70% ethanol twice for 30 s. The resulting CWR were dried *in vacuo* overnight at 30 °C. Five milligrams of CWR was used to determine lignin content using acetyl bromide method. CWR were incubated in a shaking incubator for 2 h at 50 °C with 200 μL of 25% (V:V) of acetyl bromide glacial acetic acid (VWR International, Brisbane, CA) and then diluted to 1 mL with glacial acetic acid prior centrifugation. In a new 1.5-mL tube, 100 μL was mixed to 500 μL of glacial acetic acid and 300 μL of 0.3 m sodium hydroxide was added, followed by 100 μL 0.5 m hydroxylamine hydrochloride, and between each steps, samples were mixed. An aliquot of the sample was withdraw and transferred in a UV-star microplate (Greiner Bio-One North America, Inc; Monroe, NC) and mixed to 1 volume of glacial acetic acid prior measuring the absorbance at 280 nm. The absorption coefficient used was 15.69/L g/cm (Foster *et al*., 2010) and adjusted to the pathlength based on the volume and the microplate wells (height of the liquid in the well).

### Cell wall pretreatments and saccharification

Ball-milled senesced stems (5 mg) of wild type, homozygote *c4h* mutant, *c4h* + *pVND6::C4H* and *c4h* + *pVND6::C4H-pIRX8::NST1* plants were transferred into a 2-mL screw-cap tubes and mixed to 200 μL of water or 175 μL of NaOH (1%, w/v) for hot water or dilute alkaline pretreatments, respectively, incubated at 30 °C for 30 min and autoclaved at 120 °C for 1 h. After cooling down at room temperature, samples pretreated with dilute alkaline solutions were neutralized with 2.5 N HCl (25 μL). Saccharification was initiated by adding 300 μL of 83 mm sodium citrate buffer pH 6.2 containing 133 μg/mL tetracycline, 4.4% w/w cellulase complex NS50013 and 0.44% w/w glucosidase NS50010 (Novozymes, Bagsværd, Denmark). After 24, 48 or 96 h of incubation at 50 °C with shaking (800 r.p.m.), samples were centrifuged (20 000 ***g***, 3 min) and 10 μL of the supernatant was collected for reducing sugar measurement using the DNS (3,5-dinitrosalicylate) assay (Miller, 1959). DNS reagent was prepared by dissolving 1 g of 3, 5-dinitrosalicylate in 50 mL of water at 40 °C, followed by the addition of 30 g of KNa tartrate tetrahydrate and 1.6 g of NaOH subsequently, finally the volume was adjusted to 100 mL final and the buffer was kept in dark. The DNS reaction was performed by mixing 10 μL of sample to 90 μL of DNS reagent in a PCR tube followed by incubation at 95 °C for 6 min in a PCR machine to perform the colorimetric reaction. Reducing sugars were quantified by measuring the absorbance at λ_540_ and using glucose solutions as standards.

## Supporting information

**Figure S1** Correlation of *VND6* promoter activity with the lignin biosynthesis pathway.

**Figure S2** Representation of the lignin biosynthesis pathway

**Figure S3** Expression analysis of *C4H* and *NST1* genes in stems from the cell wall engineered lines.

**Figure S4** Saccharification efficiency of biomass derived from cell wall engineered plants.

**Table S1** Cell wall thickness of fibre cells.

**Table S2** Stem diameters and densities.

**Appendix S1** Supporting experimental procedures.
