# Supplementary material for: Engineering secondary cell wall deposition in plants
Source: Plant Biotechnol J. 2012 Nov 12;11(3):325–35. doi: 10.1111/pbi.12016 (PMC3644865; doi:10.1111/pbi.12016)
Supplement: Supplementary file 1 [file pbi0011-0325-SD1.pdf]

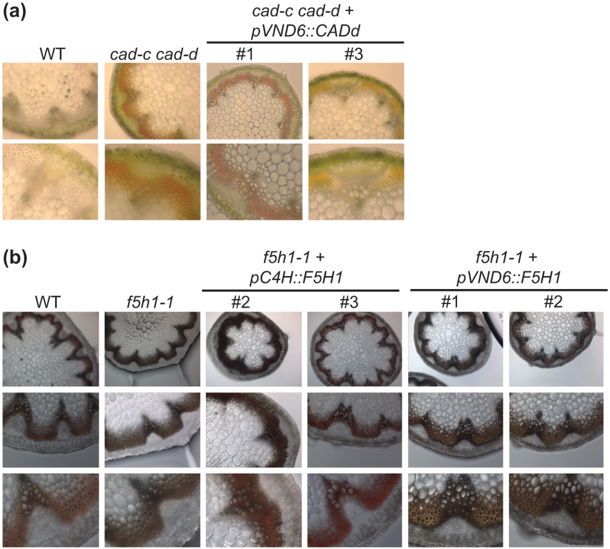

Figure S1. Correlation of *VND6* promoter activity with the lignin biosynthesis pathway. (a) Images of stem cross-sections from wildtype (WT), *cad-c cad-d* mutant, and two *cad-c cad-d* + *pVND6::CADd* complemented lines (1, 3) under bright light (b) Images of stem cross-sections from wildtype (WT), *f5h1-1* mutant, two *f5h1-1* + *pVND6::F5H1* complemented lines (1, 2), and two *f5h1-1* + *pC4H::F5H1* complemented lines (2, 3) under bright light after Mäule staining.

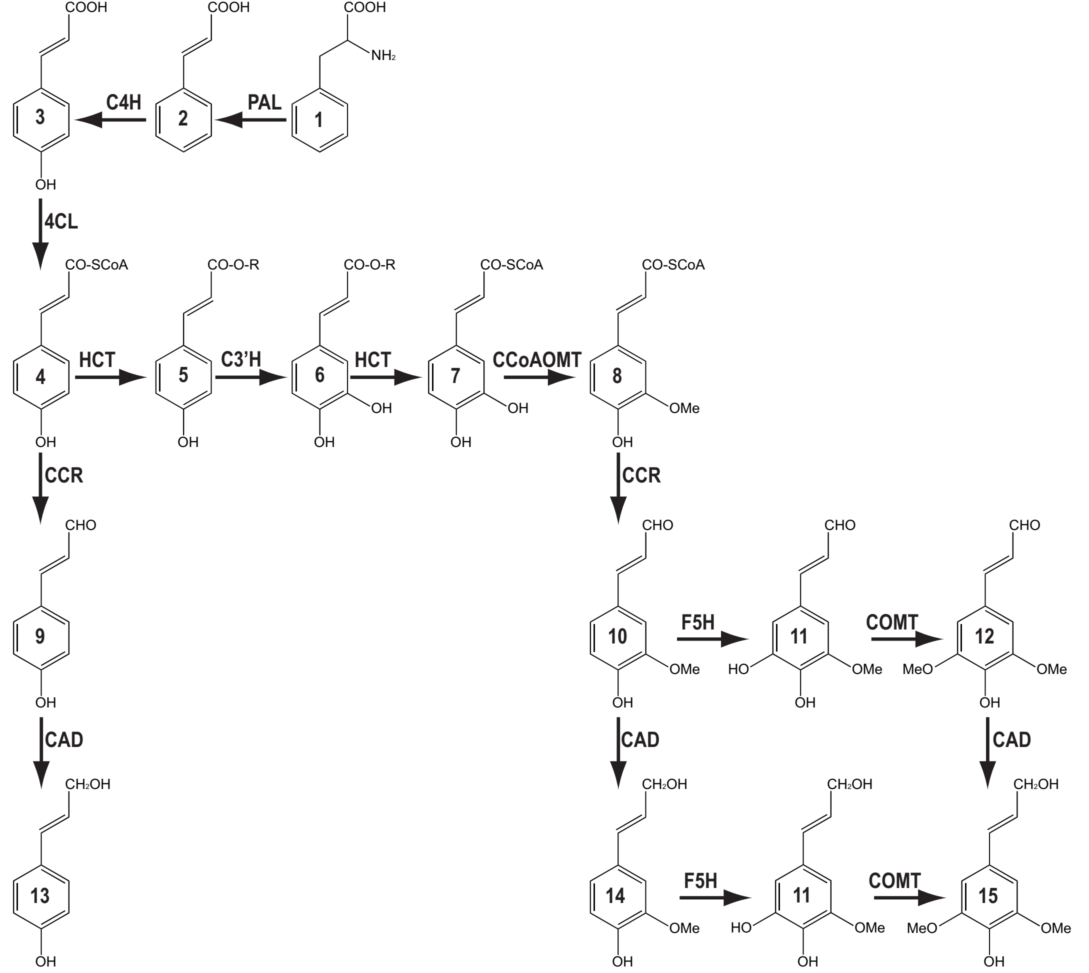

Figure S2. Representation of the lignin biosynthesis pathway

Modified lignin biosynthesis pathway from Fraser and Chapple (2011). Enzymes description, PAL: phenylalanine ammonia-lyase; C4H: cinnamate-4-hydroxylase; 4CL: 4-hydroxycinnamate CoA-ligase; HCT: hydroxycinnamoyl-CoA shikimate/quinate hydroxycinnamoyltransferase; C3'H: 4-hydroxycinnamate 3-hydroxylase; CCoAOMT: caffeoyl-CoA O-methyltransferase; CCR: hydroxycinnamoyl-CoA NADPH oxidoreductase; COMT: caffeate O-methyltransferase; CAD: hydroxycinnamyl alcohol dehydrogenase; F5H: ferulate 5-hydroxylase

Name of the lignin precursors, 1: Phenylalanine; 2, cinnamate; 3: p-coumarate; 4: p-coumaroyl-CoA; 5: *p*-coumaroyl-shikimate/quinate (R= shikimate/quinate); 6: caffeoyl-shikimate/quinate; 7: caffeoyl-CoA; 8: feruloyl-CoA; 9: p-coumaraldehyde; 10: coniferaldehyde; 11: 5-hydroxy- coniferaldehyde; 12: sinapaldehyde; 13: p-coumaryl alcohol; 14: coniferyl alcohol; 15: sinapyl alcohol

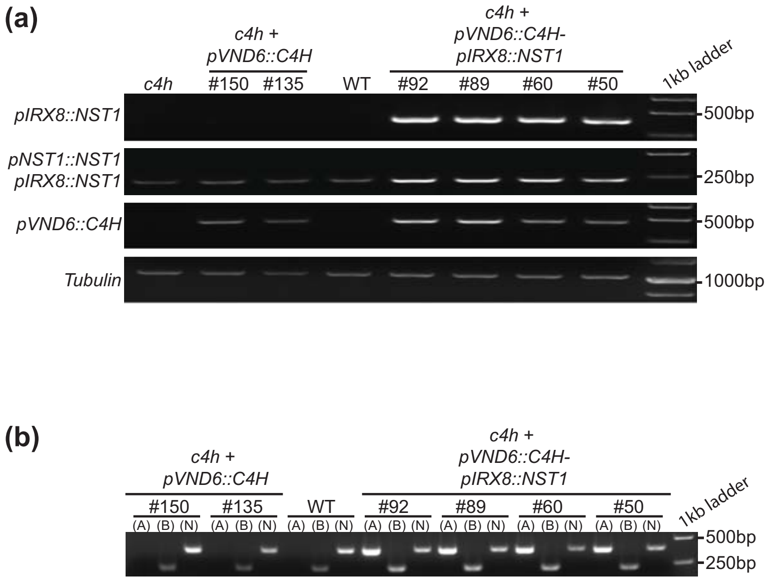

Figure S3. Expression analysis of *C4H* and *NST1* genes in stems from the cell wall engineered lines.
(a) Expression of the pVND6::C4H, pIRX8::NST1 and NST1 genes were analyzed via RT-PCR in stems from wildtype (WT), *c4h* mutant, two *c4h* + *pVND6::C4H* lines (135, 150), and four *c4h* + *pVND6::C4H* lines harboring the APFL *pIRX8::NST1* construct (50, 60, 89, 92). The tubulin gene was used as loading control.
(b) Comparative expression of both *NST1* alleles via RT-PCR, in stems from wildtype (WT), c4h + pVND6::C4H lines (135, 150), and *c4h* + *pVND6::C4H* lines harboring the APFL *pIRX8::NST1* construct (50, 60, 89, 92); (A) corresponds to the expression of the APFL *pIRX8::NST1* only; (B) corresponds to the cumulative expression of the native *NST1* and *pIRX8::NST1* genes;
(N) corresponds to the expression of the native *NST1* genes.

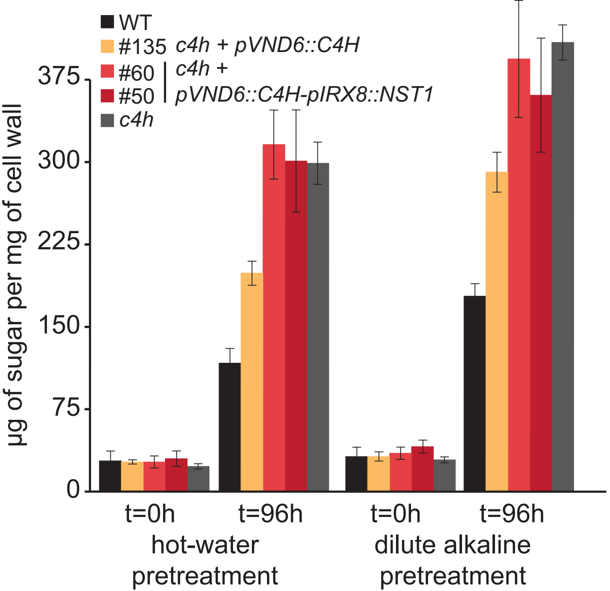

Figure S4. Saccharification efficiency of biomass derived from cell wall engineered plants. Amount of sugars released after 96h enzymatic digestion from hot-water or dilute alkali pretreated mature stems of wildtype (WT), *c4h* mutant, *c4h* + *pVND6::C4H* lines (135), and two *c4h* + *pVND6::C4H* lines harboring the APFL *pIRX8::NST1* construct (50, 60).
