# Supplementary material for: Engineering secondary cell wall deposition in plants
Source: Plant Biotechnol J. 2012 Nov 12;11(3):325–35. doi: 10.1111/pbi.12016 (PMC3644865; doi:10.1111/pbi.12016)
Supplement: Supplementary file 2 [file pbi0011-0325-SD2.docx]

| **Table S1.** Cell wall thickness of fiber cells from stem of wildtype (WT), *c4h + pVND6::C4h* and *c4h + pVND6::C4h-pIRX8::NST1* plants Asterisks indicate significant differences from the *c4h + pVND6::C4h* line using t-test  (*P < 0.01; **P < 0.001) | | | | |
| --- | --- | --- | --- | --- |
| Plant line | Average (µm) | Stdev | Number of cells measured |  |
| WT | 1.950* | 0.337 | 25 |  |
| *c4h + pVND6::C4h* (line 135) | 1.700 | 0.280 | 25 |  |
| *c4h + pVND6::C4h-pIRX8::NST1* (line 60) | 2.294** | 0.535 | 21 |  |
| *c4h + pVND6::C4h-pIRX8::NST1* (line 89) | 3.566** | 0.950 | 25 |  |

| **Table S2.** Stem diameters and densities of wildtype (WT), *c4h + pVND6::C4h* and *c4h + pVND6::C4h-pIRX8::NST1* plants (n=20). Asterisk indicates significant differences from the *c4h + pVND6::C4h* line using t-test (*P < 0.02) | | | | |  |
| --- | --- | --- | --- | --- | --- |
| Plant line | Diameter (mm) | Stdev | Density  (mg/cm^3^) | Stdev | |
| WT | 2.43* | 0.12 | 134.90 | 17.27 | |
| *c4h + pVND6::C4h* (line 135) | 2.09 | 0.13 | 140.80 | 16.58 | |
| *c4h + pVND6::C4h-pIRX8::NST1* (line 60) | 2.17 | 0.23 | 157.62* | 23.69 | |
| *c4h + pVND6::C4h-pIRX8::NST1* (line 89) | 2.11 | 0.13 | 155.07* | 12.07 | |
