# Supplementary material for: Engineering secondary cell wall deposition in plants
Source: Plant Biotechnol J. 2012 Nov 12;11(3):325–35. doi: 10.1111/pbi.12016 (PMC3644865; doi:10.1111/pbi.12016)
Supplement: Supplementary file 3 [file pbi0011-0325-SD3.docx]

**Methods S1** Supporting experimental procedures

**Generation of *pA6-pVND6-GW, pTkan-pIRX8-GW* and *pA6-pC4H-GW***

The *VND6* (At5g62380)*, IRX8* (At5g54690) and *C4H* (At2g30490) promoters corresponding to respectively 2757bp, 1577bp and 2897 bp region upstream of the ATG start codon were amplified from genomic DNA with the following primer pairs *F3-pVND6-KpnI/R3-pVND6-SpeI, F-FYpIRX8NotI-5/R-FYpIRX8AvrII-3* and *F-pREF3-5/R-FYpREF3AvrII-3* respectively and each fragment was cloned into pBlunt (Life Technologies, Grand Island, NY) to create *pBlunt-pVND6, pBlunt-pIRX8* and *pBlunt-pC4H*.

To generate the binary vectors, a Gateway cloning cassette was inserted between *HindIII* and *AvrII* restriction sites of the binary vector *pCAMBIA 1390* (accession no. AF234307) to generate a *pA6-GW* vector. The *VND6* and *C4H* promoters digested from *pBlunt-pVND6* with KpnI/SpeI and from *pBlunt-pC4H* with HindII/AvrII respectively and then inserted respectively between KpnI/AvrII and KpnI/AvrII restriction sites of the binary vector *pA6-GW* vector to generate respectively the *pA6-pVND6-GW* and *pA6-pC4H-GW* binary vectors. Finally, the *pTKan-pIRX8-GW* binary vector was generated by digesting the *IRX8* promoter from *pBlunt-pIRX8* with NotI/NheI, and then inserted between ApaI/SpeI restriction sites of *pTKan-GW* vector (Eudes et al., 2012)

**Generation of *pA6-pVND6::CADd, pA6-pVND6::F5H1* and *pA6-pC4H::F5H1***

The *CADd* (CAD5; At4g34230; Sibout et al., 2005) and *F5H1* (At4g36220; Ruegger and Chapple, 2001) encoding DNA sequences were amplified from *Arabidopsis* cDNA using gene specific primers by carrying gateway b1 and b2 recombination sites. Both pairs *F-CADd-GWb1/R-CADd-GWb2* and *F-F5H1-GWb1/R-F5H1-GWb2* were used respectively to clone *CADd* and *F5H1* encoding sequences using Gateway technology (Life Technologies, Grand Island, NY). The DNA fragments were introduced into the *pDONR221-f1* entry vector (Lalonde et al., 2010) by BP recombination to create *pDONR-F1-CADd* and *pDONR-F1-F5H1*, and then were transferred in *pA6-pVND6-GW* and *pA6-pC4H-GW* by LR recombinant reaction to create *pA6-pVND6::CADd* and *pA6-pVND6::F5H1* and *pA6-pC4H::F5H1* respectively.

**Plant material and growth conditions**

Wildtype *Arabidopsis thaliana* plant (ecotypes Columbia and Wassilewskija), *cad-c cad-d*  (ecotype Wassilewskija; Sibout et al., 2005) and *f5h1-1* (CS25128; ecotype Columbia; Ruegger et al., 1999) mutants and complemented lines were grown on soil from 8-10 old seedling germinated on soil or on sterile media (PhytoTechnology Laboratories, Shawnee Mission, KS). The complemented *cad-c cad-d* lines ( *cad-c cad-d* + *pVND6::CADd*) were generated via floral dipping with *Agrobacterium* *tumefaciens* GV3101 strain harboring the *pA6-pVND6::CADd* of homozygote *cad-c cad-d* mutants (Clough and Bent, 1998). The complemented *f5h1-1* lines (*f5h1-1* + *pVND6::F5H1*) were generated via floral dipping with *Agrobacterium* *tumefaciens* GV3101 strain harboring the *pA6-pVND6::F5H1* of homozygote *f5h1-1* mutants. Selection of T1 and T2 lines plants was made on Murashige and Skoog vitamin supplemented with 1% sucrose, 1.5% agar, and containing 40 µg/mL hygromycin.

Plants designated for analysis were grown on soil under short day condition for 4 weeks (10h:14h / light:dark cycle) prior being transferred to long day growth condition (14h:10h / light:dark cycle) until maturity at 150 µmol/m^2^/s, 22 °C, and 60% humidity. All the other plants were grown under long day condition (14h:10h / light:dark cycle) at 100 µmol/m^2^/s, 22 °C, and 55% humidity.

**Plant genomic DNA extraction**

A small leaf sample (approx 3-5 mm in diameter) was frozen in liquid nitrogen in a 1.5 mL eppendorf safelock tube containing 2 metal beads (3 mm). Frozen samples were grounded after 5 sec vortex prior adding 600 µL of CTAB buffer (0.15M Tris-HCL, 1M NaCl, 15mM EDTA, 2% Cetyltrimethylammonium bromide and 2% β-mercaptoethanol) and were incubated 65 °C in a shaker incubator at 1400rpm for 30-60min. After incubation, sampled wee cooled down to room temperature prior adding 600µL of chloroform followed by vigorous shaking, then samples were centrifuged at 20000g for 10 min at room temperature. Four hundred µL of the supernatant were withdrawn and transferred in a new tube containing 800 µL of ethanol. Samples were mixed, centrifuged for at 20000g for 10 min at room temperature and the DNA pellets were washed once with 70% ethanol prior being resuspended in 100µL TE.

**RNA extraction and RT-PCR**

Total RNAs (1 µg) were extracted using the Plant RNeasy extraction kit (Qiagen, Valencia, CA) and reverse-transcribed using the Transcriptor First Strand cDNA Synthesis Kit (Roche Applied Science, Indianapolis, IN). The obtained cDNA preparation was quality-controlled for PCR using *tub8*-specific primers *F-RT-Tub8/R-RT-Tub8* and used for the detection of *C4H* from *pVND6::C4H*, *F-RT-C4H/R-RT-GWb2*; *NST1* from *pIRX8::NST1*, *F-RT-NST1/R-RT-GWb2*; NST1; and both *NST1* (*pIRX8::NST1* and native *NST1*), *F-RT-NST1/R-RT-NST1*.

**Primers list**

| Primer name | Sequence |
| --- | --- |
| *F-RT-C4H* | GGAGACTCTTCGTCTGAGAATGGCG |
| *R-RT-GWb2* | ACCACTTTGTACAAGAAAGCTGGGT |
| *F-RT-NST1* | CGAAAGCCCTAACAGTCAGGCAATC |
| *R-RT-NST1* | GCTGGGGTACGGAGATCGGACG |
| *F-RT-Tub8* | GGGCTAAAGGACACTACACTG |
| *R-RT-Tub8* | CCTCCTGCACTTCCACTTCGTCTTC |
| *F-ref3-2* | TTCCGTATCATGTTCGATAG |
| *R-ref3-2* | AATGTCAATTTCCCAAAATC |
| *F1-pVND6* | CAAATTGCCACATTGCAGAA |
| *R1-C4H* | CGACGAGATTACGGTGGTTGA |
| *F1-pIRX8* | ACAAGATTCAGATTCATCGGTTTAG |
| *R1-NST1* | GCAGTGGCTCTGTTAGTTCTCG |
| *F-C4H-GWb1* | ggggacaagtttgtacaaaaaagcaggcttcATGGACCTCCTCTTGCTGGA |
| *R-C4H-GWb2* | ggggaccactttgtacaagaaagctgggtcACAGTTCCTTGGTTTCATAACG |
| *F-NST1-GWb1* | ggggacaagtttgtacaaaaaagcaggcttcATGATGTCAAAATCTATGAGC |
| *R-NST1-GWb2* | ggggaccactttgtacaagaaagctgggtcTCCACTACCATTCGACACGTG |
| *F-CADd-GWb1* | ggggacaagtttgtacaaaaaagcaggcttcATGGGAATAATGGAGGCAGAG |
| *R-CADd-GWb2* | ggggaccactttgtacaagaaagctgggtcAGCGTCGAGATTGCTTCCTTC |
| *F-F5H1-GWb1* | ggggacaagtttgtacaaaaaagcaggcttcATGGAGTCTTCTATATCACAA |
| *R-F5H1-GWb2* | ggggaccactttgtacaagaaagctgggtcAAGAGCACAGATGAGGCGCGT |
| *F3-pVND6-KpnI* | cccgggtaccTCCTTTACGATGTTGTTATGGGTTA |
| *R3-pVND6-SpeI* | cccgactagtGTGTGCGAGACTTTGGATTTGATCTTTTTAATTTTA |
| *F-FYpIRX8NotI-5* | cccggcggccGCACGAGCTGACTTGTACCGATGAGC |
| *R-FYpIRX8AvrII-3* | cccgcctaggCGAAGAGGGAAACTGGATCTTACG |
| *F-pREF3-5* | CGGAATGAGAGACGAGAGCAA |
| *R-FYpREF3AvrII-3* | cccgcctaggGGGCGAGAGTAATTGAAAGCAG |

**REFERENCES**

Clough, S.J. and Bent, A.F. (1998) Floral dip: a simplified method for Agrobacterium-mediated transformation of Arabidopsis thaliana. *Plant J* **16**, 735-743.

Eudes, A., George, A., Mukerjee, P., Kim, J.S., Pollet, B., Benke, P.I., Yang, F., Mitra, P., Sun, L., Cetinkol, O.P., Chabout, S., Mouille, G., Soubigou-Taconnat, L., Balzergue, S., Singh, S., Holmes, B.M., Mukhopadhyay, A., Keasling, J.D., Simmons, B.A., Lapierre, C., Ralph, J. and Loque, D. (2012) Biosynthesis and incorporation of side-chain-truncated lignin monomers to reduce lignin polymerization and enhance saccharification. *Plant Biotechnol J* **10**, 609-620.

Lalonde, S., Sero, A., Pratelli, R., Pilot, G., Chen, J., Sardi, M.I., Parsa, S.A., Kim, D.-Y., Acharya, B.R., Stein, E.V., Hu, H.-C., Villiers, F., Takeda, K., Yang, Y., Han, Y.S., Schwacke, R., Chiang, W., Kato, N., Loque, D., Assmann, S.M., Kwak, J.M., Schroeder, J., Rhee, S.Y. and Frommer, W.B. (2010) Frontiers: A Membrane Protein/Signaling Protein Interaction Network for Arabidopsis Version AMPv2. *Frontiers in Plant Physiology* **1:24**, doi: 10.3389/fphys.2010.00024

Ruegger, M. and Chapple, C. (2001) Mutations that reduce sinapoylmalate accumulation in Arabidopsis thaliana define loci with diverse roles in phenylpropanoid metabolism. *Genetics* **159**, 1741-1749.

Ruegger, M., Meyer, K., Cusumano, J.C. and Chapple, C. (1999) Regulation of ferulate-5-hydroxylase expression in Arabidopsis in the context of sinapate ester biosynthesis. *Plant Physiol* **119**, 101-110.

Sibout, R., Eudes, A., Mouille, G., Pollet, B., Lapierre, C., Jouanin, L. and Séguin, A. (2005) Cinnamyl alcohol dehydrogenase-C and -D are the primary genes involved in lignin biosynthesis in the floral stem of Arabidopsis. *Plant Cell* **17**, 2059-2076.
